# Packaging Design Using Mustard Seeds as a Natural Antimicrobial: A Study on Inhibition of *Pseudomonas fragi* in Liquid Medium

**DOI:** 10.3390/foods9060789

**Published:** 2020-06-16

**Authors:** Nur Alim Bahmid, Jenneke Heising, Vincenzo Fogliano, Matthijs Dekker

**Affiliations:** 1 Food Quality and Design Group, Wageningen University and Research, P.O. Box 17, 6700 AA Wageningen, The Netherlands; nur.bahmid@wur.nl (N.A.B.); jenneke.heising@wur.nl (J.H.); vincenzo.fogliano@wur.nl (V.F.); 2 Agriculture and Forestry Faculty, Universitas Sulawesi Barat, Majene 91412, Indonesia

**Keywords:** mustard seeds, allyl isothiocyanate, *Pseudomonas fragi*, spoilage bacteria, microbial inhibition, shelf life, antimicrobial packaging

## Abstract

*Pseudomonas fragi* is the dominant spoilage organism in various foods, especially in spoiled milk, fish, and meats. Its growth can be inhibited by releasing allyl isothiocyanate (AITC) from ground mustard seeds in food packages. This paper aims to investigate the antimicrobial potential of ground mustard seeds against *P. fragi* growth and the effectiveness of released AITC concentration from mustard seeds on microbial inhibition of the spoilage bacteria growing in the liquid medium. The AITC concentration in the headspace and the liquid medium was measured and the growth of *P. fragi* in the liquid medium was monitored. Depending on the concentration of AITC, not only growth was inhibited but a reduction of the total count of *P. fragi* was even observed. The inactivation rate (k) of *P. fragi* was estimated using first-order inactivation kinetics and the minimum gaseous-released AITC to inactivate *P. fragi* was determined. Higher AITC concentration in the headspace and liquid medium was observed when using a higher amount of ground mustard seeds and a lower food to headspace ratio. Increasing the amount of ground mustard seeds (>100 mg per 10 mL liquid medium) led to full inactivation of *P. fragi* in 48 hours. By using an inhibition sigmoid E_max_ model, the minimum gaseous-released AITC for inactivation of *P. fragi* in 48 hours was observed around 15 µg/L headspace. These results indicate that inhibition of the spoilage bacteria and extending the shelf life using ground mustard seeds is only possible by applying a careful design of the packaging system.

## 1. Introduction

Considerable amounts of fresh food are lost during processing, storage, and distribution due to spoilage in the supply chain. Microbial spoilage of food and the resulting food waste continue to be a major sustainability concern. Spoilage bacteria can quickly grow at food surfaces [1] and cause off-odors and off-tastes so the deteriorated food is considered to be unacceptable to consumers from a sensory point of view, and finally wasted. This wasted food could be minimized by modifying packaging that either extends the food shelf life or helps customers to decrease food waste [2]. Antimicrobial packaging concepts are currently being developed for extension of food product shelf life. These antimicrobial packages are able to release antimicrobial agents, which might inactivate and/or prevent the growth of spoilage bacteria on the food products.

*Pseudomonas fragi* is the dominant spoilage bacteria and has the strongest spoilage potential in a variety of foods that are stored under aerobic conditions [3,4]. It can be found in fresh fish, fresh milk, milk products, and meat [5]. Compared to other species of *Pseudomonas spp.*, this *Pseudomonas* species is the predominant bacteria that contribute significantly to food spoilage under aerobic refrigeration, followed by *Pseudomonas lundensis* and *Pseudomonas fluorescens. P. fragi* is a psychrotrophic and gram-negative bacterium that can grow at temperatures between 2 and 35 °C [6]. Many studies have found *P. fragi* to be responsible for food spoilage in foods under aerobic, vacuum, and modified atmosphere conditions [3,6,7], but there is no specific information about food preservation strategies with the help of antimicrobial packaging, such as the use of antimicrobial substances that can be added to the package and then released into packaging headspace. The release of antimicrobial compounds from a package has been proven to effectively inhibit the growth rate of microorganisms and extend the shelf life of foods [8].

Allyl isothiocyanate (AITC) is a volatile compound showing strong antimicrobial activity and bacteriostatic effects on a wide variety of spoilage bacteria by attacking the cell membranes of the bacteria [9,10,11]. AITC penetrates into the cells to disrupt the cytoplasmic membrane of bacteria. The AITC penetration leads to cell leakage of their intracellular compounds and disaggregation of cell walls which causes loss of the integrity of the cellular structure [12,13,14]. This effective mechanism of bacterial inhibition has been investigated and reported to be able to inhibit the microbial growth of spoilage bacteria in packaged food by adding the AITC inside the packaging. Kanemaru and Miyamoto [15] monitoring the growth of spoilage bacteria, e.g., *P. fragi*, for 24 h in nutrient broth containing mustard extracts or/and pure AITC found that 0.043% of mustard extract and 3.6 ppm of pure AITC were sufficient to inhibit the growth of *P. fragi* in the broth for 24 h. Pang, et al. [16] reported AITC combined with modified atmosphere packaging (49% CO_2_/0.5% O_2_/50.5% N_2_) inhibited the growth of *P. aeruginosa* by extending the shelf life of the catfish fillets from about 12 to 27 h (18 µg/L) and 41 h (36 µg/L) at 20 °C. Another form is the usage of mustard oil showing its antifungal activity against foodborne mold strains in the liquid with the minimum concentration values ranging from 0.8 to 50 ppm [17]. However, the antimicrobial effect on the spoilage bacteria was reported on the amount of added AITC/mustard extracts in the packaging system or into the bacterial medium, not on the actual gaseous concentration in the headspace exposed to the food. Furthermore, no literature related to the minimum gaseous concentration of AITC inhibiting *P. fragi* growth was found, whereas the *P. fragi* was identified to be the dominant bacteria, with an incidence between 56.7% and 79.0% on spoiled meat [3]. The effectiveness of AITC on inhibition and reduction of *P. fragi,* therefore, needs to be further investigated to quantify the minimum headspace concentration of AITC required to inhibit the bacteria.

The release rate of AITC from natural sources, e.g., mustard seeds, can be increased by manipulating the properties of the sources [18]. Mustard seeds contain sinigrin, a glucosinolate acting as a precursor for AITC formation [19]. Once the cells of mustard seeds are damaged and hydrated, myrosinase hydrolyzes the sinigrin and then AITC is formed and released [20]. Our previous study reported that a higher release of AITC was observed in mustard seeds ground into smaller sizes and with lower fat content, however, the capacity to control bacterial growth was not investigated [21]. In this study, we explored the antibacterial potential of the ground mustard seeds against *P. fragi* simulating a food pack system and using brain heart infusion (BHI) broth as an example of highly perishable food. To assess the antimicrobial activity of ground mustard seeds against *P. fragi*, the effects of volume ratio of the liquid medium to headspace and temperature on the released AITC concentration in the headspace and the liquid medium were monitored in relation to design a packaging system that effectively inhibits *P. fragi* in the food system. The effectiveness of headspace AITC was also investigated by the determination of the minimum concentration to inhibit and inactivate the *P. fragi* in a liquid medium. These results provide valuable insight for the customers and the development of an antimicrobial packaging concept with mustard seeds that can be applied to a variety of food products.

## 2. Materials and Methods

### 2.1. Chemicals

Mustard seeds (*Brassica juncea*) were purchased from Natuurproduct.com, Jacob Hooy brown mustard seeds. Plate count agar (PCA) was from Merck KGaA. Brain heart infusion (BHI) broth powder was from Oxoid LTD. Disposable inoculation loops were from VWR International. Glycerol was from Fisher Scientific. Diethyl ether was from sigma Aldrich. Sterile Cryovial was from Simport Scientific and Peptone physiological salt solutions (PFZ) were from Tritium Microbiology.

### 2.2. Preparation of Ground Mustard Seeds

Mustard seeds were freeze-dried (Martin Christ) for 5 days and then immediately stored in a desiccator for 3 h. The freeze-dried seeds were ground by using a milling machine (Analysette 3 Pro ball mill, Fritsch group) with an amplitude of 2.2 mm. Afterward, the ground seeds were sieved (Retsch) to obtain the size ranges: 200–315 µm and 600–800 µm. The ground seeds (25 g) were completely defatted for 6 h by soxhlet extraction [22] using diethyl ether solvent that has a low boiling point (35 °C) to avoid the inactivation of myrosinase. The ground defatted seeds were collected and put in a desiccator for 1 day to remove any water or solvent left in the seeds to prevent the pre-hydrolysis of sinigrin due to moisture uptake. Finally, the ground seeds were stored in a freezer at −20 °C until usage.

### 2.3. Preparation of Plates, Media, and Culture

#### 2.3.1. PCA-Plate and BHI Broth Preparation

To count log_10_ CFU/mL per mL of sample, non-selective PCA was used. PCA was prepared using a 2 liter Duran flask filled with 1 liter of MilliQ water and 22.5 g of PCA powder. The flask was then heated while being stirred. After the powder was completely dissolved, these flasks were sterilized at 121 °C for 15 min. Afterward, the flasks were cooled down to approximately 45 °C in a water bath (Salm en Kipp). The cooled down flask was brought in the biosafety cabinet and then disinfected with 70% ethanol. About 10 mL of liquid PCA-agar was poured into Petri dishes and left to dry. Finally, the Petri dishes were stored in a refrigerator at 5 °C until usage for the microbial experiments. BHI broth was prepared to be used as a growth medium (liquid medium). BHI broth powder (37 g) was dissolved with Milli-Q water (1 L) at a Duran flask. The solution was then sterilized in an autoclave at 121 °C for 15 min. Afterward, the broth was cooled down to room temperature. Finally, the broth was stored in a refrigerator at 5 °C until usage for the experiments.

#### 2.3.2. Stock and Bacterial Strain of *P. fragi* Culture Preparation

A bacterial strain of *P. fragi* culture Gruber 1905 (DSM 3456) obtained from the Food Microbiology Group at Wageningen University and Research was used. With a disposable inoculation loop (VWR International), a colony from the pure *P. fragi* culture was taken and suspended into 9 mL of sterilized BHI broth in a 12 mL Greiner tube. The tube was then incubated in an incubator (IKS) at 30 °C for 48 h. To prepare a stock culture of *P. fragi*, the incubated BHI broth (0.7 mL) containing inoculated bacteria was mixed 0.3 mL of glycerol in a 1.2 mL sterile Crovial. The glycerol was added to protect the bacteria from freeze damage, e.g., membrane leakage [23]. Finally, the culture was stored in a freezer at −20 °C till usage.

A suspension of *P. fragi* culture was prepared 12 h in advance of the microbial experiments. A tube with 1 mL of stock culture was taken out of the freezer and suspended into a 12 mL Greiner tube containing 9 mL of sterile BHI broth. The tube was incubated in a stove at 30 °C for 12 h. Afterward, the suspension was diluted to approximately 5 Log_10_ CFU/mL in pre-manufactured 9 mL peptone physiological salt solutions. The suspension was plated out on PCA plates in triplicate. The plates were incubated for 48 h at room temperature. After incubation, three randomly chosen colonies from each plate were tested to confirm that the colonies were indeed *P. fragi* colonies. The presence of the enzymes oxidase and catalase were both tested and a Gram stain test was performed.

### 2.4. Determination of Allyl Isothiocyanate in Both the Headspace and the Liquid Medium, and Total Bacteria of the P. fragi

**Figure 1:**
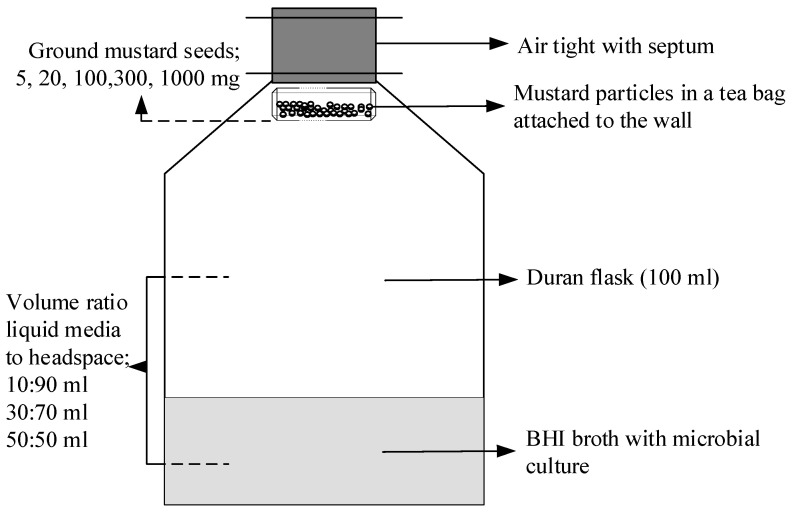
Schematic picture of the Allyl isothiocyanate (AITC) release system used for measuring the headspace AITC and the growth of *P. fragi* in the brain heart infusion (BHI) broth (liquid medium).

A closed system was used as a packaging simulation system to investigate the AITC release in the headspace and the antimicrobial effect of AITC on the growth of *P. fragi* (Figure 1). In this experiment, three different liquid medium to headspace ratios were investigated; (1) 10:90 (10 mL broth: 90 mL headspace), (2) 30:70 (30 mL broth: 70 mL headspace) and 50:50 (50 mL broth: 50 mL headspace). Five different amounts of ground mustard seeds were used; 0 (control), 5, 20, 100, 300, 1000 mg. For 1000 mg ground seeds, this amount was initially used for preliminary experiments to investigate the effect of the sizes (200–315 and 600–800 µm) of ground seeds and temperatures (4 and 20 °C). The system was designed in an airtight system using a Duran flask (100 mL volume) and a cap with an airtight septum. Using these caps, samples of the broth (0.1 mL) could be taken out by a needle and syringe which can then be inserted for headspace AITC measurement without the possibility of the gas to escape from the flask. In the disinfected biosafety flow cabinet, sterile Duran flasks were filled with 9, 27, and 45 mL of pure BHI broth (control) and BHI broth inoculated with the suspension of *P. fragi* (1, 3, and 5 mL) containing approximately 5 Log_10_ CFU/mL. The ground seeds (5, 20, 100, 300, and 1000 mg) covered by sterile tea bags were completely submerged for 5 s and then immediately placed in the Duran flasks closed with septum lid. The broth/headspace samples were then stored in a refrigerator at 4 °C and in a dark shelf at room temperature (20 °C). To prevent contamination, the flasks, the tea bags, the Greiner tubes containing the liquid medium, pipette tips, and water were first sterilized at 121 °C for 15 min and disinfected with 70% ethanol before the start of the experiment.

#### 2.4.1. AITC Measurement in the Headspace and the Liquid Medium

The concentration of volatile AITC in the headspace and the liquid medium of the closed system was measured at 6, 24, 48, 72, and 96 h. For headspace measurement, the headspace of the samples was injected manually with Solid Phase Micro Extraction (SPME) (100 µm polydimethylsiloxane, red fiber 23ga) fiber for 1 min and the used SPME was then injected into the GC-FID.

For liquid AITC in the liquid medium, AITC was measured by a liquid injection method modified from Marton and Lavric [24]. The broth (0.1 mL) was taken out from the flask and then added into hexane (1.5 mL) in Eppendorf tubes. The mixtures were vortexed for 2 min and then centrifuged at 2627 g at 20 °C for 5 min. The solution was filtered by using a PTFE 45 um filter (phenomenex) into the brown HPLC vials. Finally, the samples were measured by GC-FID in conjunction with 10 µL syringe cemented needle (Hamilton Microliter), connected to an autosampler (Thermo-Scientific, Waltham, MA, USA, TriPlus Autosampler).

During the AITC measurement in headspace and liquid, a Restek Rxi-5HT GC column (30 m, 0.25 mm internal diameter, 0.25 μm stationary film thickness) was utilized. The inlet temperature was 250 °C, a splitless mode for 1 min (10 mL/min flow) was maintained. The initial temperature of the oven was 40 °C during the first minute of running, the temperature was increased until 280 °C with a rate of 10 °C per minute. The runtime was 26 min and helium gas was used as a carrier (1 mL/min). The detector had a temperature of 270 °C, with a flow of 350 mL air and 35 mL H_2_. AITC was analyzed with Xcalibur software, in which AITC is known to be detected as two separate peaks [25]. The calibration was quantified using pure AITC in concentrations 1 to 1000 ppm dissolved in hexane. This calibration was used to quantify the AITC concentration in the liquid medium. For the headspace AITC concentration, the SPME values were converted by using an experimentally determined relation between the SPME and the direct injection of headspace samples and calibration with known amounts of AITC in hexane using liquid injection.

#### 2.4.2. Microbial Count of *P. fragi* Growth23

The number of cells N (CFU/mL) surviving in the liquid medium was monitored at 6, 24, 48, 72, 96, 120, 144, and 168 h. In addition, the number of cell decrease during 48 h were also observed in every 3 h. The broth (1 mL) was taken from each Duran flask using a sterile syringe and needle passing through the septum without releasing AITC out of the flask. After serial dilution in 9 mL PFZ tubes, 100 μL of each dilution was plated out on PCA plates. Using this plate, the detection limit for *P. fragi* colony was 2 log_10_ CFU/mL. The plates were incubated at 37 °C for 48 h and then colonies were counted. All plates containing at least 20 colonies to a maximum of 300 colonies were used in the calculations for the Log_10_ CFU/mL determination. The oxidase, catalase, and gram tests were performed on three random selected colonies per growth medium in order to confirm the presence of *P. fragi*.

### 2.5. Survival Curves Modeling

The concentration of AITC and the number of bacterial cells were measured in triplicate for each sample and the data were presented as mean and standard deviation of three replicates using Microsoft office 2016. To describe the kinetics of the microbial survival from the obtained experimental data, first-order inactivation kinetics [26] were used:

(1) $$S\left(t\right)=10^{\left(-\frac{t}{D}\right)}$$

(2) $$Log\;S\left(t\right)=-\frac{t}{D}$$

where *S (t)* = N/N_0_ is the survival fraction being N and N_0_ the number of microorganisms at time t and time zero, respectively and the level of inactivation, log_10_ *S (t)*. D is the decimal reduction time (the time needed to reduce the numbers/concentration by one log_10_):

(3) $$D=\frac{ln10}{k}=\frac{2.303}{k}$$

in which k is a first-order rate constant (h^−1^).

Microsoft Excel was used to perform the estimation of the parameter, the rate constant (k) with the Equation (3) substituted into Equation (2). The experimental data on the number of cells of *P. fragi* observed within 48 h were fitted properly modeled using the solver in Microsoft Office Excel 365 ProPlus. Equation (2) was best fitted to the data set by minimizing the sum of the squared differences between the empirical data and the fitted values provided by Equation (2) using the Solver add-in [27].

## 3. Results

### 3.1. The Effect of Particle Size and Temperature on AITC Release in the Headspace and the Antimicrobial Effect against P. fragi

**Figure 2:**
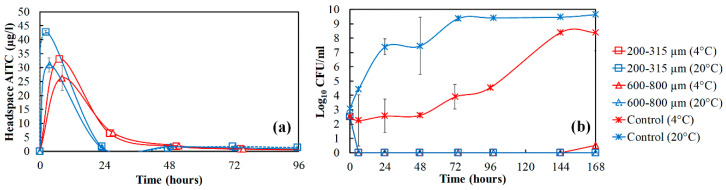
(**a**) The release of Allylisothiocyanate (AITC) from ground mustard seeds with different particle sizes (200–315 and 600–800 µm) stored at different temperatures (4 and 20 °C) for 7 days; (**b**) the antimicrobial effect of AITC against *P. fragi* from the released AITC.

The preliminary results of the AITC released from mustard ground seeds (1000 mg) and its antimicrobial effect on the growth of *P. fragi* with the effect of particle size and temperature are shown in Figure 2. The temperature has a clear effect on AITC stability in the headspace and the particle size shows an effect only in the shortest times. In Figure 2a, the higher AITC concentration at 20 °C was observed at a few hours after rehydration. A higher temperature could initially increase the AITC volatility, which then causes an increase of the driving force of AITC from the ground seeds to headspace [28]. However, the concentration was shifted after 24 h where the higher AITC was observed at 4 °C. These results demonstrate that the temperature is crucial for the stability of AITC needed to prolong bacterial growth inhibition.

In Figure 2b, the antimicrobial effect of AITC against *P. fragi* was observed. The initial bacterial count of *P. fragi* was around 3 log_10_ CFU/mL. In the control (sample without ground seeds), *P. fragi* grew quickly and the bacterial population reached the stationary phase after 48 h at 20 °C, compared to the control sample at 4 °C taking 144 h to reach the maximum growth. The presence of AITC released from the ground seeds at 4 and 20 °C completely inactivated the bacterial cells in the liquid medium within 6 h, the bacteria were no longer detectable. In an extended period (two weeks), the *P. fragi* growth was not observed in the BHI broth. These results indicate that the concentration of AITC released from 1000 mg ground seeds into the headspace kills all the *P. fragi*. Therefore, for the next experiment, lower amounts of ground seeds were studied to evaluate the antimicrobial effect for the particle size (200–315 µm) and temperature (20 °C).

### 3.2. The Effect of Ground Mustard Seeds and the Liquid Medium to Headspace Ratio on AITC Concentration

**Figure 3:**
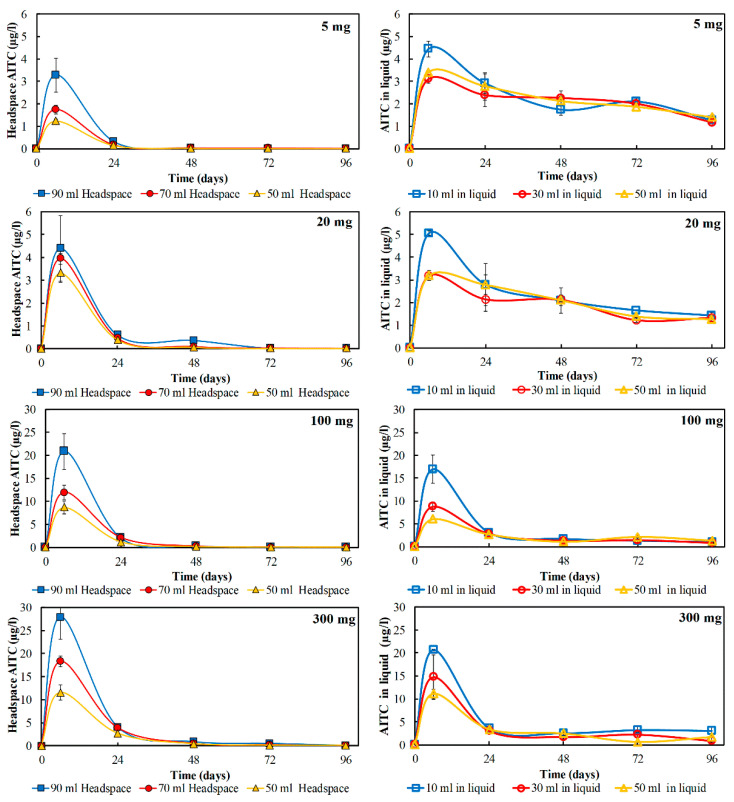
The concentration of Allyl isothiocyanate (AITC) in the headspace (90, 70, and 50 mL) (left side) and the liquid medium (10, 30, and 50 mL) (right side) with an increase of amount of ground mustard seeds (5, 20, 100, 300 mg) added into the closed system.

Figure 3 shows the concentration of AITC released from different amounts of added ground mustard seeds in the closed containers containing different volumes of the liquid medium. A reduction in the AITC concentration occurred in the presence of fewer ground seeds. In the headspace, AITC concentration was apparently decreased in the higher liquid medium to headspace ratio (Figure 3). About 27 µg/L AITC was released from 300 mg of ground mustard seeds into 90 mL of headspace at 6 h, while only 10 and 20 µg/L was released into 50 and 70 mL of packaging headspace. These results indicate that besides the fat content and particle sizes of ground seeds [21], AITC release into headspace is also influenced by the food to headspace volume ratio in the packaging system.

The liquid medium was a better phase to keep AITC remaining in higher concentration rather than in the headspace. In Figure 3, the AITC in both headspace and liquid medium reached peaks at 6 h. Afterward, the headspace AITC dropped down to almost undetectable level after 24 h, while at the same time the AITC in the liquid medium reduced to about 3 µg/L that was above the headspace concentration. This concentration was declined only by 1 µg/L to 7 days. It can be concluded that AITC stability was also influenced by the phase where AITC partitions.

### 3.3. The Effect of Ground Mustard Seeds on the Growth of P. fragi Inoculated in the Different Volumes of the Liquid Medium

**Figure 4:**
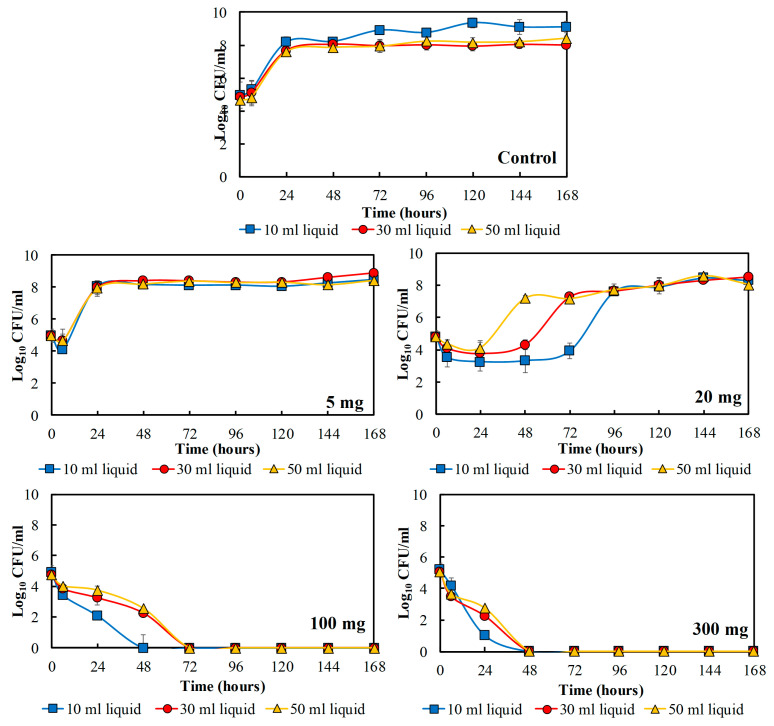
The total bacterial count of *P. fragi* for 7 days influenced by the amount of ground mustard seeds added into the package and the liquid medium volume present in the package.

Figure 4 shows the total bacteria of *P. fragi* affected by the amount of ground seeds and the ratio of liquid medium to headspace volume in the packaging system. The total bacteria of *P. fragi* was dependent on the amount of ground mustard seeds in the closed system; more ground seeds in the system speeded up the inhibition process of the bacteria. Figure 4 shows a complete microbial inactivation in 48–72 h with the highest amounts of ground seeds (100 and 300 mg) in the package setup. The use of the smaller liquid medium to headspace ratio speeded up the inactivation rate of *P. fragi* as clearly observed with 100 mg ground seeds. In 10 mL of BHI liquid medium, 100 mg of ground seeds were able to completely inactivate the bacteria in 48 h, but in 30 and 50 mL liquid medium, the bacteria population was reduced to be the undetectable level after around 72 h.

On the other hand, 5 mg of ground mustard seeds only reduced the bacterial population by around 1 log_10_ CFU/mL before *P. fragi* grew as fast as the control samples, reaching the maximum total bacteria after 24 h. Ground seeds (20 mg) first reduced the population of *P. fragi* by around 1–3 log_10_ CFU/mL and subsequently also prolonged the lag phase before the bacteria started to regrow. The growth of *P. fragi* was postponed longer for smaller liquid medium to headspace ratio. *P. fragi* inoculated in the 10 mL, 30 mL, and 50 mL of liquid medium, *P. fragi* started to grow again after 1,2 and 3 days of incubation time, respectively.

### 3.4. The Inactivation Rate Constant of Inoculated P. fragi within 48 h of Contact Time with AITC

**Figure 5:**
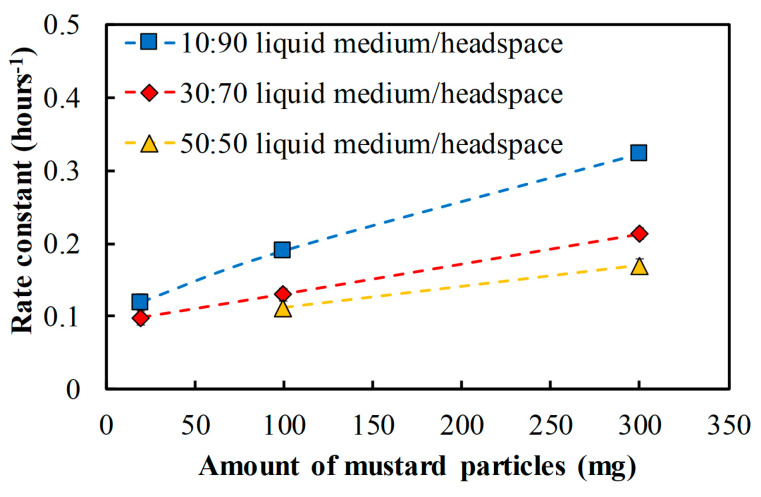
Estimated microbial inactivation rate constant (k) using first-order inactivation kinetics for different amounts of ground mustard particles and liquid medium to headspace ratio.

**Figure A1:**
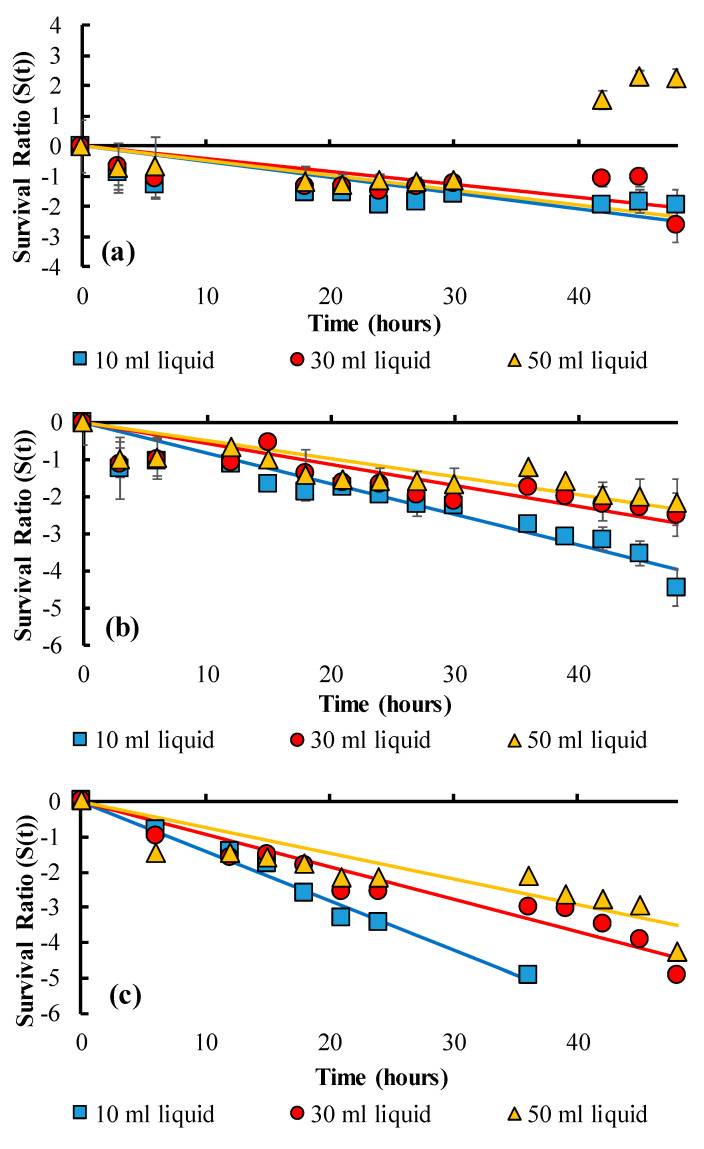
The kinetic microbial inactivation with (**a**) 20, (**b**) 100 and (**c**) 300 mg of ground mustard seeds on the P. fragi inoculated in different liquid medium volume.

Figure 5 shows the inactivation rate constant, a parameter estimated by first-order inactivation kinetics model (Equation (2)) to mathematically describe the kinetic microbial inactivation for 48 h (Figure A1 in Appendix A). The results clearly show that the larger amount of ground mustard seeds present in the packaging system increased the rate constant k. Consequently, the time needed to reduce the bacterial count by one decimal log_10_ becomes shorter (lower D-value). In Figure 5, with 20 mg ground seeds, the inactivation rate constant of *P. fragi* at 10 mL of liquid medium solution was around 0.13 h^−1^. With 300 mg ground seeds, the rate constant increased 2.5 fold (0.33 h^− 1^) leading to a reduction of the total bacterial count to an undetectable level after 48 h (Figure 4). These estimated rate constants decreased with an increase of liquid medium to headspace ratio. For 20 mg ground seeds and 50 mL liquid medium, the rate constant cannot be estimated because the first order inactivation model did not fit with the observed data due to bacterial regrowth after 24 h (Figure A1 in Appendix A). These results clearly show that the ratio of food to headspace volume for inoculating the bacteria and the amount of added ground seeds in the packaging system take an important role in the reduction and inactivation of the bacteria.

### 3.5. The Fits of the Inhibitory Sigmoid E_max_ Model to the Highest Concentration-Inhibition Curve

**Figure 6:**
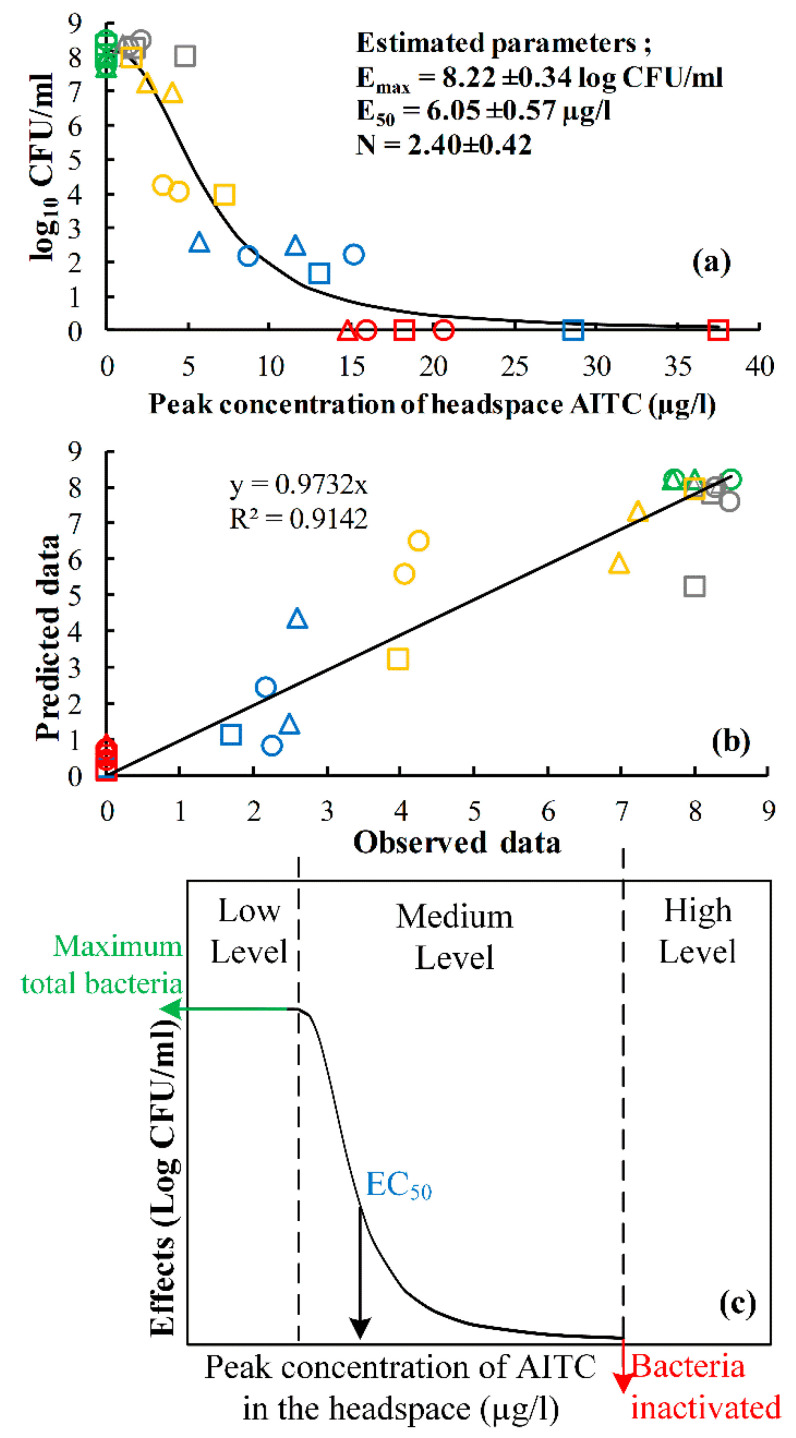
(**a**) the total bacteria of *P. fragi* at 48 h as a function of peak concentration of AITC in the headspace, followed by inhibitory sigmoid E_max_ and the estimated parameters, (**b**) The goodness of fit of the E_max_ model, and (**c**) schematic overview of inhibitory E_max_ model. The different shape shows different volumes; square (10 mL), circle (30 mL), triangle (50 mL) and different colors depict different amount of mustard seeds; red (300 mg), blue (100 mg), yellow (20 mg), grey (5 mg), and green (control).

In this study, the highest (peak) concentration of AITC at 6 h shown in Figure 3 can be linked to the total bacteria of *P. fragi* (initially inoculated ±5 log_10_ CFU/mL) in order to determine the minimum headspace concentration to effectively inactivate the bacteria in 48 h. The highest concentration-inhibition curve can be described using a modified inhibitory sigmoid E_max_ model (Equation (4)) [29,30] as shown in Figure 6a,

(4) $$E=E_{0}-\frac{C_{6h}{}^{N}\;E_{max}}{C_{6h}{}^{N}+EC_{50}{}^{N}}$$

where *E* is the total bacteria (log_10_ CFU/mL) at 48 h showing the effect of the highest concentration of gaseous AITC; *E_0_* is the baseline total bacteria (log_10_ CFU/mL) without AITC (0 µg/L); *E*_max_ is the maximum effect (log_10_ CFU/mL) of the highest concentration of gaseous AITC, which assumed to be equal to *E*_0_ due to full inactivation; *EC*_50_ is the highest concentration of gaseous AITC causing half of *E*_max_; *N* is slope factor (hill factor) that determines the steepness of the concentration-inhibition curve; *C*_6*h*_ is the highest (peak) concentration of AITC at 6 h.

The inhibitory sigmoid E_max_ model fitted well with the highest concentration-inhibition curve. As shown in Figure 6b, the estimated parameters provide low standard deviations with no correlation amongst the parameters. Furthermore, high goodness of fit (*R^2^* = 0.93) was obtained from this model. It indicates the inhibitory sigmoid E_max_ model accurately described the relation of AITC with the total bacteria and can be employed to determine the minimum gaseous released AITC to inactivate the bacteria. The headspace minimum concentration estimated by using the model is discussed below.

## 4. Discussion

In this work, different AITC concentrations in the headspace and liquid medium were evaluated, with varying the amounts of ground seeds and the ratio of liquid medium to headspace volume. As expected the concentration of released AITC increased with the amount of ground seeds present in the packaging system. The higher AITC concentration was also observed in the decreased liquid medium volume. The smaller volume of liquid medium absorbed less AITC from the headspace causing higher concentrations for the lower ratio of liquid medium-to-headspace volume. Upon partitioning of AITC to the smaller volume of liquid medium, the concentration of AITC became higher, but the amount of AITC will be less. Therefore, it can be concluded that in a food pack system if less food is present the concentration in both food and headspace will be higher.

**Figure A2:**
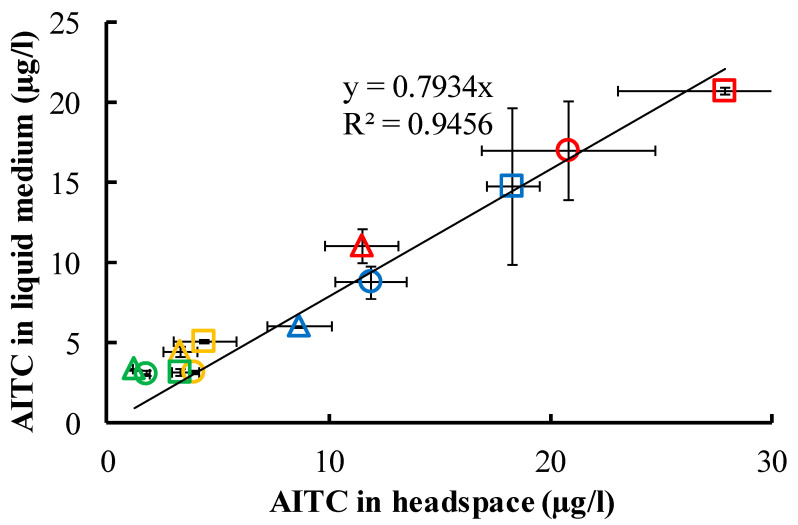
The relation of Allyl isothiocyanate (AITC) release from ground mustard seeds in the headspace and in the liquid medium at 6 h. The different shape shows different volumes; square (10 mL), circle (30 mL), triangle (50 mL) and different colors depict different amount of mustard seeds; red (300 mg), blue (100 mg), yellow (20 mg) and green (5 mg).

The bacterial growth and inhibition depend on the concentration of antimicrobial compounds in the headspace and the liquid medium in the package. The relation between the antimicrobials and growth inhibition was described by Clemente, et al. [17] that the increasing mustard oil (to 7 ppm) inhibited almost 50% of the growth of *R. stolonifer,* and the complete inhibition concentration was at almost 15 ppm. Comparing the effectiveness of AITC between both gaseous and aqueous phases, Several studies show that AITC is less effective to inhibit the bacteria in the (model) food phase compared to the gaseous phase [9,12]. Lin, et al. [12] claimed the better bactericidal activity of AITC vapor due to (a) better penetration of gaseous AITC into the food, (b) low water solubility of AITC and high volatility of AITC and (3) AITC degradation in the aqueous phase. The claim was based on the comparison of antibacterial activity of AITC in gaseous (indirect contact with food) versus liquid (direct contact with food) form against the bacterial growth on food, in which AITC was more effective at a lower concentration in the gaseous form, compared to the liquid form. On the other hand, no study reports a comparison of AITC concentration in the packaged food and in the headspace to determine whether microbial inhibition occurred because of the gaseous AITC or AITC in the food. In our study, the actual AITC concentration in the headspace and liquid medium was daily measured for a few days. The result shows that the AITC was released from ground mustard seeds into the headspace, then partitioned into the food. In Figure A2 in Appendix A, AITC concentration in the headspace and the liquid medium at 6 h have a linear correlation, but the AITC concentration was slightly higher in the headspace than AITC in the liquid medium. The higher AITC in the headspace does not mean that headspace AITC gave a better effect on microbial inhibition against *P. fragi*, because the liquid medium also contains AITC. Instead, the headspace AITC might have a direct effect on the bacterial growth on the surface, while in the food the AITC should first partition from the headspace into the liquid medium to then inhibit the bacteria in the liquid medium [31] by attacking the growing cells of *P. fragi* over time. In addition, the observed better effect of gaseous AITC against the spoilage bacteria is observed for aerobic bacteria [32]. The aerobic bacteria normally grow on the surface part of the packed food because of the dependence on oxygen availability. For these reasons, the gaseous AITC is more effective to inhibit the spoilage bacteria on the surface of the packed food.

The first-order inactivation kinetic model (Equation (2)) describes well the inactivation of *P. fragi* for 48 h. From the model, the rate constant (k) parameters ware estimated as shown in Figure 5. The minimum inactivation rate (k) to fully inactivate the total bacteria (inoculated at ±5 log_10_ CFU/mL) for 48 h is over 0.15 h^−1^, which was determined from 100 and 300 mg of ground mustard seeds that totally reduced the total bacteria (Figure 3). In agreement with our results, although no results on the inactivation rate were given, microbial inactivation in the presence of AITC at over 20 °C was reported by Guo, et al. [33]. This study reported a fully reduced population from 5 logs *L. innocua* in Tryptic soy broth (TSB) at 22 °C with an edible coating containing 2 to 4% of AITC and 1% of AITC for 2 and 3 days of inactivation, respectively. These indicate the AITC has a strong antimicrobial activity to inactivate the total population of spoilage bacteria in the (model) food and the inactivation rate is faster at higher AITC concentration in the packaging system.

The total bacteria is highly related to the concentration of antimicrobial compounds added to the headspace of food packaging [14]. The initial concentration of pure compound added into packaging at 0 h can be assumed to be considered equal with the concentration peak (after a few hours) of antimicrobial compounds released from the naturally antimicrobial carrier added into the packaging system. To determine the headspace AITC concentration that is sufficient to inhibit the *P. fragi*, the peak headspace concentration-inhibition curve was fitted with the inhibitory sigmoid E_max_ model (Equation (4)) as shown in Figure 6. The model gives an understanding of the headspace concentration of AITC released from mustard seeds required to reduce and inactivate the bacteria in packed food products. Figure 6a or Figure 6c defines the level of peak AITC concentration that can affect the total bacteria. There are three levels of concentration; low, medium, and high. In the low level, the concentration resulted in no effect in total bacteria. The medium level can be defined as a sensitive concentration because the AITC reduces the total bacteria, but the bacteria might still re-grow after reduction. In the high level, the concentration induced a complete reduction or inactivation of the bacteria. From this model, the minimum-gaseous released AITC causing inhibition and inactivation of *P. fragi* in 48 h can be defined, which are around ±6.05 µg/L (1.49 ppm) for microbial inhibition and at least 15 µg/L (3.70 ppm) for inactivation. The inhibitory concentrations were lower than the minimum concentration of pure AITC (3.6 ppm) initially added into the nutrient broth to inhibit the growth of *P. fragi* for 24 h [34]. Comparable minimum vapor concentrations of AITC for inactivation against other microorganisms inoculated at 4–5 log_10_ CFU in agar plates for 2 days was also observed; *Pseudomonas fluorescens* (36–50 µg/L), mold (16–22 µg/L), yeast (16–31 µg/L) [18,35]. The different results of minimum concentration might be because of the difference in experimental design, for example using agar as culture media or adding AITC directly into food products, while in our study the liquid medium was used to grow the bacteria. In summary, AITC concentration, 15 µg/L (3.70 ppm), released from mustard seeds (>100 mg) into the 90 mL headspace and 10 mL liquid medium is sufficient to totally reduce *P. fragi* in the liquid medium to an undetectable level for 48 h.

For the application of mustard seeds in a food package, the required ground mustard to inhibit the bacteria in a food, e.g., fresh catfish fillet [16], can be estimated using the first-order inactivation model to calculate the inactivation rate (k). The catfish fillet initially contained around 3.5 log_10_ CFU/g of the microbial population of *P. aeruginosa* [16]. To inhibit the total bacteria *P. aeruginosa* in 50 mg of catfish fillet for 48 h, the calculated k was observed, around 0.168 h^−1^. In relation to Figure 5, the obtained k indicates that 300 mg of ground mustard seeds are assumed to effectively inhibit the bacteria in the catfish fillet for 48 h. Nevertheless, this AITC concentration may vary due to several other factors to be considered, e.g., particle sizes, temperatures, fat content, and food components influencing the AITC release and stability in the headspace.

The biggest challenge of mustard seeds in the application in packaged foods is the sensory effects on food products. The sensory impact highly depends on the AITC concentration in the packaged food. This sensory effect of AITC concentration has been reported in some studies. AITC concentration (0.1 to 2.5%) in hummus [20] and in kimchi [36] was organoleptically accepted by the panelists. Lopes, et al. [37] also evaluated sensory the AITC concentration (0.5 to 2.5 µL/L) on Brazil peanuts, in which the highest doses (2.5%) of AITC did not change the sensory properties of Brazil peanuts. In this study, we used 20 to 300 mg of ground mustard seeds in 100 mL packaging volume (0.02 to 0.3% *w/v*) releasing around 1–30 µg/L in the headspace and 3–20 µg/L in the liquid medium, which is a lower concentration than those in the aforementioned literatures, assuming fewer effects of food sensory. However, regardless of high dependence on the type of food, sensory evaluation needs to be performed for further confirmation of gaseous AITC concentration acceptable by consumers.

## 5. Conclusions

The aim of this study was to explore the antimicrobial potential of ground mustard seeds and the effectiveness of released AITC concentration from mustard seeds on microbial inhibition of *P. fragi* growing in the liquid medium. The concentration of AITC in the headspace and the liquid medium is dependent on the amount of ground mustard seeds added into the packaging system; more ground seeds released more AITC in the headspace and liquid medium. Consequently, the higher the concentration of AITC the stronger the inhibition of *P. fragi* growth. The AITC released from 100 mg of ground seeds in the package with 10 mL liquid medium fully inactivated the cells of *P. fragi* in 48 h. The amounts of AITC released in a package with 20 mg of ground mustard seeds did not completely inactivate the bacteria but was able to effectively reduce the bacterial count by 1–3 log_10_ CFU/mL before the bacteria re-grew after 1–3 days of incubation depending on the liquid medium to headspace ratio. For a smaller liquid medium to headspace ratio, an increased AITC concentration in the headspace is required to extend the microbial inhibition of *P. fragi*. By using the sigmoid E_max_ inhibition model, the minimum-released AITC concentrations required to inhibit *P. fragi* are determined, around ±3 µg/L (0.74 ppm) for reduction and 15 µg/L (3.70 ppm) for full inactivation. These results show that ground mustard seeds are effective to control the spoilage by *P. fragi* on food products at a concentration not influencing the food sensory. This study shows the effectiveness of AITC released from ground mustard seeds to control the spoilage bacteria, thereby giving insight for customers in the design of a food packaging system to extend shelf life of a variety of packaged food products.
